# Adjuvant radiotherapy for locally advanced upper tract urothelial carcinoma

**DOI:** 10.1038/srep38175

**Published:** 2016-12-02

**Authors:** Yun-Ching Huang, Ying-Hsu Chang, Kuo-Hsiung Chiu, Alan W. Shindel, Chia-Hsuan Lai

**Affiliations:** 1 Division of Urology, Department of Surgery, Chang Gung Memorial Hospital at Chiayi, Taiwan; 2 Graduate Institute of Clinical Medical Sciences, College of Medicine, Chang Gung University, Taoyuan, Taiwan; 3 Division of Urology, Department of Surgery, Chang Gung Memorial Hospital at Linko, Taiwan; 4 Nursing Department, Chang Gung Memorial Hospital, Chiayi, Taiwan; 5 Volunteer Faculty, Department of Urology, University of California at San Francisco, USA; 6 Genomic Health Inc., Redwood City, USA; 7 Department of Radiation Oncology, Chang Gung Memorial Hospital at Chiayi, Taiwan

## Abstract

There is relatively little literature on adjuvant radiotherapy after radical nephroureterectomy with bladder cuff excision (RNU) for patients with upper tract urothelial carcinoma (UTUC). This study was designed to determine the efficacy of adjuvant radiotherapy for patients with pT3N0M0 UTUC. We retrospectively reviewed 198 patients treated with RNU between December 2001 and January 2015. Postoperative radiotherapy was administered in 40 (20.2%) of patients. Patients who received radiotherapy were younger than those that did not (65.2 vs. 70.5 years, *p* = 0.023). With median follow up of 29.1 months, Kaplan-Meier analysis with the log-rank test demonstrated no significant differences between those omitting vs receiving adjuvant radiotherapy in regards to 2-year rates of overall survival (72.0% vs. 73.4%, *p* = 0.979), cancer-specific survival (73.2% vs. 75.3%, *p* = 0.844), and recurrence-free survival (61.2% vs. 66.3%, *p* = 0.742). However, in multivariable analysis with Cox regression, young age, absence of chronic kidney disease, negative lymphovascular invasion, negative surgical margin, and adjuvant chemotherapy were also associated with better cancer-specific survival. In conclusion, adjuvant radiotherapy did not offer any significant benefit in terms of overall, cancer-specific, and recurrence-free survivals in patients with pT3N0M0 UTUC after RNU. More effective systemic adjuvant chemotherapy is necessary to improve the outcome of these patients.

Urothelial carcinoma (UC) is the second most common cancer of the genitourinary tract^1^. UC may occur in the upper urinary tract (renal pelvis and ureter) or lower urinary tract (bladder and urethra). Upper tract urothelial carcinoma (UTUC) accounts for just 5% of UC in United States but up to 30% of cases of UC in Taiwan^1^,^2^. There is a possible association of UTUC with exposure to arsenic in drinking water, Chinese herbal medications containing with aristolochic acid (AA), and blackfoot disease in Taiwan^3^,^4^,^5^. Although UTUC are infrequent, they tend to have a worse prognosis than UC of the bladder as 78% of UTUC is invasive at the time of diagnosis^6^.

Radical nephroureterectomy with bladder cuff excision (RNU) is the gold standard for high-risk UTUC regardless of the tumor location in upper urinary tract^7^. Local-regional control of pT1 and pT2 stage disease is excellent in surgical series and isolated local recurrence is rare^8^. However, in the setting of pT3N0M0 UTUC a high rate of recurrence and distant metastases has been reported even after RNU^9^. The prognosis of recurrent and distant metastasis UTUC is poor with or without salvage therapy^10^.

Due to the relative rarity of the condition, the efficacy of postoperative adjuvant therapies for patients with pT3N0M0 UTUC is not well defined. The aim of this study was to retrospectively evaluate clinical outcomes of pT3N0M0 UTUC patients receiving adjuvant radiotherapy after radical surgery. We hypothesized that adjuvant radiotherapy after RNU for UTUC would be associated with superior overall, cancer-specific, recurrence-free, locoregional disease-free and metastasis-free survival.

## Results

### Patient population

Clinical data from a total of 198 pT3N0M0 patients who underwent radical nephroureterectomy and bladder cuff excision were analyzed. From this population 40 (20.2%) underwent adjuvant radiotherapy following surgery whereas 158 (79.8%) patients did not receive radiotherapy. The median age for the overall study cohort was 68.6 years (range 23.6 to 91.6 years). There was a slight female predominance (52.0%).

Patients in radiotherapy group were significantly younger than patients in the non-radiotherapy group (65.2 vs 70.5 years, *p* = 0.023). There was no significant difference in gender, current smoking status, American Society of Anesthesiologists (ASA) score, recurrent bladder tumor, recurrent contralateral UTUC, estimated glomerular filtration rate (eGFR), chronic kidney disease (CKD), tumor grade, lymphovascular invasion (LVI), carcinoma *in situ* (CIS) and positive surgical margin between patients who did or did not receive adjuvant radiotherapy (Table 1).

### Radiotherapy dose and acute toxicity

The median dose of radiotherapy was 50.4 Gy (range 23.4 to 64.8 Gy). Thirty-five (87.5%) patients completed the scheduled radiotherapy protocol. Acute toxicity was evaluated in all of the 40 patients and occurred in 34 (85.0%). However, toxicity was grade 1–2 in 31 of these 40 patients (77.5%). Three patients developed grade 3 acute toxicity but only one completed the scheduled radiotherapy protocol. Platinum based adjuvant chemotherapy was administered in 21 (52.5%) patients of the radiotherapy group including gemcitabine and cisplatin (6), and cisplatin, fluorouracil and leucovorin (15) regimens. A mean of 4 cycles (range 2 to 6 cycles) were administered (Table 2).

### Survival and Recurrence

The median follow-up duration for the whole cohort after surgery was 29.1 months (range 6.4 to 164.9 months). At the end of follow-up, 66 (33.3%) patients had died due to cancer-related causes and 13 (6.6%) patients had died from other causes. Overall 79 (39.9%) patients had recurrence of UTUC in the entire cohort; of these, 18 (9.1%) patients had isolated locoregional failure, suggesting the most common recurrence pattern was distant metastasis (Table 3).

**Figure 1:**
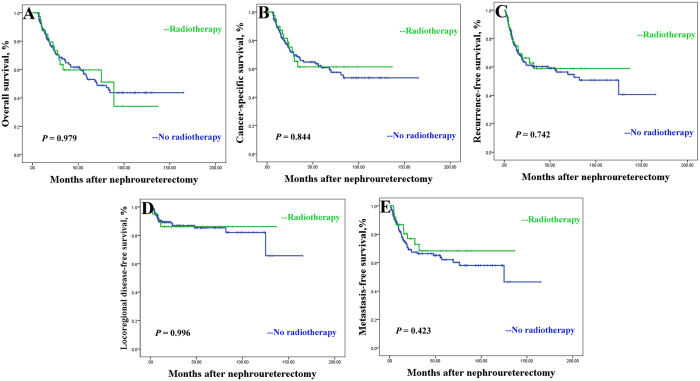
Overall survival curve of patient with (green curves) vs without (blue curves) adjuvant radiotherapy (**A**), cancer-specific survival (**B**), recurrence-free survival (**C**), locoregional disease-free survival (**D**) and metastasis-free survival (**E**).

The median survival in the group without radiotherapy was 29.0 months (range 6.4 to 164.9 months) and in the group with radiotherapy was 29.6 months (range 7.4 to 136.3 months). Between the non-radiotherapy and radiotherapy treatment groups, there were no statistically significant differences in 2 year overall (72.0% vs 73.4%, *p* = 0.979), cancer-specific (73.2% vs 75.3%, *p* = 0.844), recurrence-free (61.2% vs 66.3%, *p* = 0.742), locoregional disease-free (86.9% vs 86.2%, *p* = 0.996) and metastasis-free survivals (67.4% vs 74.4%, *p* = 0.423, Fig. 1).

Further subset analysis in the group of patients who received no adjuvant therapy, radiotherapy alone and concurrent chemoradiotherapy treatment showed a significant difference in 2 years overall survival (72.0% vs 49.5% vs 91.0%, *p* = 0.032), and cancer-specific survival (73.2% vs 52.68% vs 91.0%, *p* = 0.018). There was no statistically significant difference in 2 years recurrence-free survival (61.2% vs 48.8% vs 77.5%, p = 0.099), locoregional disease-free (86.9% vs 79.5% vs 90.5%, *p* = 0.661) and metastasis-free survivals (67.4% vs 57.7% vs 85.7%, *p* = 0.128).

On multivariate analysis with Cox hazards regression, advanced age (HR 1.43; 95% CI 1.09 to 1.88; *p* = 0.011), CKD (HR 1.64; 95% CI 1.18 to 2.28; *p* = 0.003), LVI (HR 1.79; 95% CI 1.35 to 2.37; *p* = 0.000), positive surgical margin (HR 1.76; 95% CI 1.16 to 2.67; *p* = 0.008) and adjuvant chemotherapy (HR 0.33; 95% CI 0.17 to 0.66; *p* = 0.002) were independent prognostic factor for cancer-specific survival. Gender, current smoking status, ASA score, recurrent bladder tumor, recurrent contralateral UTUC, tumor grade, CIS, and adjuvant radiotherapy (HR 0.60; 95% CI 0.27 to 1.34; *p* = 0.213) were not significantly associated with cancer specific survival (Table 4).

## Discussion

Patients with pT3 UTUC comprise an extremely high risk group for subsequent disease relapse despite aggressive radical surgery. The prognosis for recurrent and distant metastatic UTUC is poor with or without salvage therapy^10^. Therefore, there is great interest in effective adjuvant therapies that may reduce the chances of disease recurrence.

Isolated locoregional recurrence is rare and distant metastasis is the most common tumor relapsed patterns in patients with pT3 UTUC in this cohort study. Radiotherapy is traditionally considered to be a local control tool for tumors, and so there would be limitations when using radiotherapy to treat pT3 UTUC patients. Therefore, we again suggest that pT3 UTUC should be considered as a systemic nature and that perioperative chemotherapy should be recommended^11^.

However, due to the low incidence of UTUC, there is relatively little literature on adjuvant radiotherapy after RNU for patients with UTUC. The European Association of Urology (EAU) is one of the only scientific urological associations that published guidelines on UTUC. The guidelines have been updated in 2016 on the EAU web site (http://uroweb.org) and state that the role of adjuvant radiotherapy is not well defined, neither alone, nor in combination with chemotherapy^12^.

Due to the conflicting results of studies on high risk UTUC, the question of whether radiotherapy could play some role in management of the disease has been open to debate^13^. Maulard-Durdux *et al*. and Catton *et al*., similar to our study, indicated no benefits with postoperative adjuvant radiotherapy in UTUC^14^,^15^. However, Jwa *et al*. reported that adjuvant radiotherapy may be beneficial in terms of locoregional and bladder control in patients with stage III/IV UTUC^16^. Chen *et al*. showed that radiotherapy may improve overall survival for patient with T3/4 cancer of the renal pelvis or ureter and delayed bladder relapse^17^. Finally, Jang *et al*. recommended that concurrent chemoradiotherapy could improve the outcomes in patients with T3/4 and/or node positive upper tract transitional cell carcinoma^18^. There are several potential explanations for the conflicting results between studies. Most studies to date have enrolled small and heterogeneous populations. In the largest series to date, Chen *et al*. identified 133 patients (pT1–4 and/or N+); however just 52 (39.1%) of these patients had pT3 or pT4 UTUC and almost 19% patients had residual disease following surgery^17^. Another large series by Jwa *et al*. reported 127 patients with stage III/IV UTUC but the mean follow-up period in the radiotherapy group was shorter than that of the surgery-alone group (27.0 vs 44.3 months)^16^. Further characterization of the patient population revealed a significantly higher positive surgical margin, LVI, systemic chemotherapy, and presence of high grade tumor in the radiotherapy group than in the no radiotherapy group.

Our study does not support the use of radiotherapy in management of pT3 UTUC. However, similar to Jang *et al*., our study does report some benefit from combination chemotherapy and radiation^18^. Whether the same benefit can be obtained from chemotherapy alone is a compelling question for future research.

Our current study included only patients with pT3N0M0 UTUC; we are unaware of any published data that is entirely focusing on single stage of high risk patient in evaluating the effect of adjuvant radiotherapy. Furthermore, all of our patients underwent RNU, which is the gold standard for high-risk UTUC regardless of the tumor location in upper urinary tract^7^. Many prior reports have included patients who did not undergo nephroureterectomy but rather had a more limited surgical resection of the kidney and ureter^14^,^15^,^16^,^17^,^18^,^19^,^20^. Taken together, we believe that ours is a study to better understand optimal management of patients with pT3 UTUC.

In Western countries UTUC is more common in men, with a male-to-female ratio of 2:1^10^. However, the current study demonstrated that a slight female predominance of UTUC in Taiwan; this result is similar to other collaborative reports from Taiwan^21^,^22^. Many risk factors contribute to UTUC development including maintenance dialysis and AA^4^,^23^. There are slightly more women on dialysis than men in Taiwan^24^. Furthermore, the majority of Taiwanese people who use AA-containing Chinese herbal products are female^25^. These findings may drive the higher female to male prevalence ratio for UTUC in Taiwanese women compared to their Western counterparts.

This study has a number of limitations such as retrospective study, small number of patients in the radiotherapy group and difficult determination of the exact radiation technique. The radiotherapy group tended to have a higher positive surgical margin compared with non-radiotherapy group although this difference did not reach statistical significance. In addition, the effect of radiotherapy for chemotherapy remains largely unaccountable in the retrospective data collection. However, in our study, which is to our knowledge the largest to date, adjuvant radiotherapy was not significantly associated with overall, cancer-specific, recurrence-free, locoregional disease-free, and metastasis-free survival in the entire cohort or on any subset analysis from a single institution. Our findings with respect to chemotherapy are intriguing and merit closer consideration and future research.

In conclusion, in this single-institution retrospective study there did not appear to be a significant benefit to adjuvant radiation therapy for pT3 UTUC in terms of overall, cancer-specific, recurrence-free, locoregional disease-free or/and metastasis-free survival. Age, CKD, LVI and surgical margin remain important predictors of cancer-specific survival. More effective systemic adjuvant chemotherapy is necessary to improve the outcome of these patients. Prospectively randomized control study studies on the role of radiotherapy in patients with pT3 UTUC are required to better define its role.

## Methods

### Patient population

This study was approved and the need for informed consent has waived by the ethics committee of Chang Gung Medical Foundation Institutional Review Board (201600556B0). All the procedures were in accordance with the principles of the Declaration of Helsinki. From December 2001 to January 2015, we respectively reviewed patients with UTUC who were treated with nephroureterectomy with bladder cuff excision and regional lymph node dissection at our institution. The inclusion criteria were pT3N0M0 patients treated by RNU with curative intent and with no prior history of radiotherapy, and those who had at least 6 months of follow up after NU. All patients had cross sectional imaging (computerized tomography or magnetic resonance urography) and cystoscopy before surgery. Excretory urography, retrograde urography, ureteroscopy and cytology were used as indicated for additional evaluation in some patients.

Clinical features evaluated gender, age, current smoking status, ASA score, recurrent bladder tumor, recurrent contralateral UTUC, postoperative renal function, final pathology (including assessment of tumor grade, LVI, presence of CIS, and surgical margin), radiotherapy dose and acute radiotherapy toxicity (if any). Acute radiotherapy toxicities were reported based on standard Radiation Therapy Oncology Group criteria^26^. eGFR was calculated and recorded using the Modification of Diet in Renal Disease formula as 186 × (serum creatinine)^−1.154^ × (age)^−0.203^ × (0.742 if female)^27^. CKD was defined as eGFR less than 60 mL/minute/1.73 m^2^^28^. In patients who died, cause of death and time from diagnosis to death were recorded.

### Pathological evaluation

Tumor grade was determined using the 2004 World Health Organization grading system, and tumor staging was determined according to the 7^th^ edition AJCC TNM classification by urologic pathologists at our institution.

### Outcome Measures

After radical surgery, cross sectional image was performed to confirm that there was no recurrence before initiation of adjuvant radiotherapy. After completion of adjuvant therapy patients were generally followed up with physical examination, serum biochemistry, renal ultrasound and cystoscopy every 3 months during the first 2 years, every 6 months between the 3rd through 5th years, and then once every year. Chest radiography and either abdominopelvic computerized tomography or magnetic resonance urography were performed annually to assess for locoregional recurrence and metastasis. Bone scan and chest computerized tomography were performed according to physician’s judgement.

The efficacy of postoperative adjuvant radiotherapies were analyzed by stratification into two groups: group1, all cases who received nephroureterectomy with bladder cuff excision alone (Non-radiotherapy); group 2, all cases who received adjuvant radiotherapy with and without concurrent chemotherapy (Radiotherapy).

All patients were treated with 3-D conformal radiotherapy or intensity modulated radiotherapy. Radiotherapy treatment volume consisted of the tumor bed and regional lymph nodes. The total dosage of postoperative adjuvant radiotherapy was at the discretion of the treating physician and ranged from 45.0 to 64.8 Gy at 1.8–2.0 Gy in one fraction per day, 5 days per week. To meet criteria for adjuvant radiotherapy, treatment must have been started within 3 months of undergoing nephroureterectomy. For patients who elected chemotherapy, one of two cisplatin-based chemotherapy regimens was offered:

1. GC, consisting of gemcitabine (1000 mg/m^2^ on day 1 and 8) and cisplatin (70 mg/m^2^ on day 1)].
2. PFL, consisting of cisplatin (50 mg/m^2^ on day 1), fluorouracil (750 mg/m^2^ on day 1, 2, 3) and leucovorin (50 mg on day 1, 2, 3)^29^.

Four to six cycles of chemotherapy was planned according to the patient’s status. Cisplatin was replaced by carboplatin if the glomerular filtration rate was less than 40 ml/minute/1.73 m^2^.

Locoregional recurrence was defined as recurrence in the tumor bed or regional lymph nodes, and distant metastasis was defined as tumor recurrence outside this region. Survival time was calculated from the date of radical surgery to final follow-up or death (cancer-specific or from other causes).

### Statistics

Fisher’s exact with *x*^*2*^ and two-tailed *t* tests were used for comparisons between groups in categorical and continuous variables, respectively. Overall, cancer-specific, recurrence-free, locoregional disease-free and metastasis-free survival curves were derived by the Kaplan-Meier method with the log-rank test. Univariate analysis with the log-rank test and multivariate analysis with Cox hazards regression were applied to evaluate the value of prognostic factors including gender, age, current smoking status, ASA score, recurrent bladder tumor, recurrent contralateral UTUC, CKD, tumor grade, LVI, CIS, positive surgical margin, adjuvant radiotherapy, and adjuvant chemotherapy in predicting overall, cancer-specific, and recurrence-free survival. Bladder or contralateral renal pelvis or ureter recurrence were considered to be second primaries and not included in the calculation of overall, cancer-specific, and recurrence-free survival. All statistical analyses were performed using SPSS version 20 (SPSS Inc., Chicago, IL, USA). Statistical significance was set at *P* < 0.05.

## Additional Information

**How to cite this article**: Huang, Y.-C. *et al*. Adjuvant radiotherapy for locally advanced upper tract urothelial carcinoma. *Sci. Rep.* **6**, 38175; doi: 10.1038/srep38175 (2016).

**Publisher's note:** Springer Nature remains neutral with regard to jurisdictional claims in published maps and institutional affiliations.

## Figures and Tables

**Table 1:** Clinical characteristics of patients with pT3 UTUC.

	No Adjuvant Radiotherapy (*n* = 158)	Adjuvant Radiotherapy (*n* = 40)	*P* value
Gender, n (%)			1.000
Female	82 (51.9)	21 (52.5)	
Male	76 (48.1)	19 (47.5)	
Age, yrs, median (range)	70.5 (23.6–91.6)	65.2 (39.5–82.9)	**0.023**
Less than 68.6, n (%)	75 (47.5)	24 (60.0)	0.215
68.6 or greater, n (%)	83 (52.5)	16 (40.0)	
Current smoking status, n (%)			0.119
No	139 (88.0)	31 (77.5)	
Yes	17 (10.7)	9 (22.5)	
Unknown	2 (1.3)	0 (0)	
ASA score, n (%)			0.931
1	3 (1.9)	1 (2.5)	
2	70 (44.3)	19 (47.5)	
3	84 (53.2)	20 (50.0)	
4	1 (0.6)	0 (0)	
Recurrent bladder tumor, n (%)	41 (25.9)	8 (20.0)	0.540
Recurrent contralateral UTUC, n (%)	10 (6.3)	2 (5.0)	1.000
eGFR (mL/min/1.73 m^2^), median (range)	43.1 (0–86.5)	44.9 (0–77.4)	0.265
Less than 60, n (%)	132 (83.5)	33 (82.5)	0.817
60 or greater, n (%)	26 (16.5)	7 (17.5)	
Tumor Grade, n (%)			0.125
Low	11 (7.0)	0 (0)	
High	147 (93.0)	40 (100)	
Lymphovascular invasion, n (%)	31 (19.6)	9 (22.5)	0.664
Carcinoma *in situ*, n (%)	20 (12.7)	6 (15.0)	0.793
Positive surgical margin, n (%)	5 (3.2)	4 (10.0)	0.084

UTUC: upper tract urothelial carcinoma, ASA: American Society of Anesthesiologists, eGFR: estimated glomerular filtration rate.

**Table 2:** Dose and acute toxicity of radiotherapy (n = 40).

Radiotherapy dose, Gy, median (range)	50.4 (23.4–64.8)
Unknown, n (%)	1 (2.5)
Scheduled protocol, n (%)	
Complete treatment	35 (87.5)
Incomplete treatment	5 (12.5)
Acute radiotherapy toxicity, n (%)	
0	6 (15.0)
1	8 (20.0)
2	23 (57.5)
3	3 (7.5)
Adjuvant chemotherapy regimen, n (%)	21 (52.5)
Gemcitabine and cisplatin	6 (15.0)
Cisplatin, fluorouracil and leucovorin	15 (37.5)

**Table 3:** Comparison of the disease relapse pattern in the patients with pT3 UTUC.

	No Adjuvant Radiotherapy (n = 158)	Adjuvant Radiotherapy (n = 40)	*P* value
Distant metastasis, n (%)	44 (27.8)	10 (25.0)	0.843
Locoregional failure, n (%)	13 (8.2)	5 (12.5)	0.371
Locoregional + distant metastasis, n (%)*	7 (4.4)	0 (0)	0.348

^*^Indicated that the first site of failure was in the tumor bed or regional lymph nodes, and distant metastasis synchronously.

**Table 4:** Univariate and multivariate analysis for overall, cancer-specific and recurrence-free survival in the patients with pT3 UTUC.

	Overall survival				Cancer-specific survival				Recurrence-free survival			
	Univariate		Multivariate		Univariate		Multivariate		Univariate		Multivariate	
	HR (95% CI)	*P* value	HR (95% CI)	*P* value	HR (95% CI)	*P* value	HR (95% CI)	*P* value	HR (95% CI)	*P* value	HR (95% CI)	*P* value
Gender	1.21 (0.78–1.89)	0.391	1.01 (0.57–1.77)	0.981	1.39 (0.85–2.27)	0.188	1.35 (0.72–2.55)	0.353	1.25 (0.80–1.96)	0.323	1.27 (0.73–2.22)	0.403
Advanced age*	1.54 (1.22–1.94)	**0.000**	1.47 (1.14–1.88)	**0.003**	1.56 (1.21–2.01)	**0.001**	1.43 (1.09–1.88)	**0.011**	1.31 (1.05–1.65)	**0.019**	1.25 (0.98–1.60)	0.075
Current smoking	1.00 (0.73–1.38)	0.979	1.16 (0.78–1.74)	0.466	1.01 (0.71–1.44)	0.948	1.28 (0.81–2.03)	0.292	1.04 (0.75–1.45)	0.811	1.20 (0.80–1.80)	0.389
ASA score		0.972		0.798		0.730		0.255		0.225		0.120
Recurrent bladder tumor	1.40 (0.82–2.39)	0.220	1.23 (0.69–2.21)	0.477	1.19 (0.68–2.09)	0.542	1.01 (0.55–1.85)	0.986	1.02 (0.62–1.69)	0.926	1.30 (0.75–2.26)	0.344
Recurrent contralateral UTUC	1.15 (0.70–1.90)	0.586	1.08 (0.64–1.83)	0.767	1.49 (0.74–3.01)	0.270	1.50 (0.73–3.09)	0.274	1.17 (0.71–1.94)	0.533	1.23 (0.72–2.08)	0.446
CKD†	1.33 (1.00–1.77)	0.052	1.50 (1.09–2.06)	**0.012**	1.42 (1.06–1.90)	**0.019**	1.64 (1.18–2.28)	**0.003**	1.38 (1.06–1.82)	**0.019**	1.66 (1.23–2.24)	**0.001**
Tumor grade	1.32 (0.65–2.66)	0.444	1.59 (0.75–3.37)	0.224	1.22 (0.61–2.47)	0.575	1.63 (0.77–3.48)	0.205	1.12 (0.63–1.99)	0.706	1.26 (0.68–2.34)	0.457
LVI	1.64 (1.29–2.08)	**0.000**	1.69 (1.30–2.19)	**0.000**	1.68 (1.30–2.17)	**0.000**	1.79 (1.35–2.37)	**0.000**	1.55 (1.22–1.99)	**0.000**	1.63 (1.24–2.14)	**0.000**
CIS	1.40 (1.07–1.83)	**0.015**	1.19 (0.88–1.61)	0.269	1.37 (1.02–1.84)	**0.038**	1.14 (0.81–1.60)	0.450	1.46 (1.12–1.91)	**0.006**	1.37 (1.02–1.85)	**0.038**
Positive surgical margin	1.57 (1.11–2.24)	**0.012**	1.64 (1.11–2.44)	**0.014**	1.65 (1.16–2.36)	**0.005**	1.76 (1.16–2.67)	**0.008**	1.48 (1.04–2.10)	**0.027**	1.73 (1.16–2.57)	**0.007**
Radiotherapy	0.99 (0.57–1.72)	0.979	0.59 (0.29–1.23)	0.158	0.94 (0.51–1.73)	0.844	0.60 (0.27–1.34)	0.213	0.92 (0.53–1.62)	0.777	0.71 (0.34–1.50)	0.371
Chemotherapy	0.68 (0.43–1.07)	0.099	0.42 (0.24–0.74)	**0.003**	0.57 (0.32–1.01)	0.056	0.33 (0.17–0.66)	**0.002**	0.69 (0.44–1.09)	0.113	0.43 (0.24–0.76)	**0.004**

UTUC: upper tract urothelium carcinoma, HR: hazard ratio, CI: confidence interval, ASA: American Society of Anesthesiologists, CKD: chronic kidney disease, LVI: lymphovascular invasion, CIS: carcinoma *in situ*.

^*^Indicated that age exceeded the median age of the patient population, 68.6 years.

^†^Indicated that estimated glomerular filtration rate < 60 mL/min/1.73 m^2^.

## References

[b1] Siegel R., Ma J., Zou Z. & Jemal A. Cancer statistics, 2014. CA Cancer J Clin 64, 9–29, doi: 10.3322/caac.21208 (2014). 24399786

[b2] Health Promotion Administration, Ministry of Health and Welfare, Taiwan. Cancer registry annual report, 2013, Taiwan. http://www.hpa.gov.tw/Bhpnet/Web/Stat/StatisticsShow.aspx?No=201604210001.

[b3] Tan L. B., Chen K. T. & Guo H. R. Clinical and epidemiological features of patients with genitourinary tract tumour in a blackfoot disease endemic area of Taiwan. BJU Int 102, 48–54, doi: 10.1111/j.1464–410X.2008.07565.x (2008). 18445081

[b4] Chen C. H. . Aristolochic acid-associated urothelial cancer in Taiwan. Proc Natl Acad Sci USA 109, 8241–8246, doi: 10.1073/pnas.1119920109 (2012). 22493262 PMC3361449

[b5] Wang Y. H. . Comparing the joint effect of arsenic exposure, cigarette smoking and risk genotypes of vascular endothelial growth factor on upper urinary tract urothelial carcinoma and bladder cancer. J Hazard Mater 262, 1139–1146, doi: 10.1016/j.jhazmat.2012.08.056 (2013). 23009795

[b6] Margulis V. . Outcomes of radical nephroureterectomy: a series from the Upper Tract Urothelial Carcinoma Collaboration. Cancer 115, 1224–1233, doi: 10.1002/cncr.24135 (2009). 19156917

[b7] Roupret M. . European Association of Urology Guidelines on Upper Urinary Tract Urothelial Cell Carcinoma: 2015 Update. Eur Urol 68, 868–879, doi: 10.1016/j.eururo.2015.06.044 (2015). 26188393

[b8] Li C. C. . Significant predictive factors for prognosis of primary upper urinary tract cancer after radical nephroureterectomy in Taiwanese patients. Eur Urol 54, 1127–1134, doi: 10.1016/j.eururo.2008.01.054 (2008). 18243511

[b9] Wu C. F. . The impact factors on prognosis of patients with pT3 upper urinary tract transitional cell carcinoma. J Urol 178, 446–450, dicussion 450, doi: 10.1016/j.juro.2007.03.115 (2007). 17561129

[b10] Munoz J. J. & Ellison L. M. Upper tract urothelial neoplasms: incidence and survival during the last 2 decades. J Urol 164, 1523–1525 (2000). 11025695

[b11] Huang Y. C. . The Efficacy of Postoperative Adjuvant Chemotherapy for Patients with pT3N0M0 Upper Tract Urothelial Carcinoma. J Urol 194, 323–329, doi: 10.1016/j.juro.2015.03.077 (2015). 25796114

[b12] Rouprêt M. . Upper Urinary Tract Urothelial carcinoma. (Date of access: 20/10/2016). *European Association of Urology web site* http://uroweb.org/guideline/upper-urinary-tract-urothelial-cell-carcinoma/(2016).

[b13] Lucca I., Leow J. J., Shariat S. F. & Chang S. L. Diagnosis and management of upper tract urothelial carcinoma. Hematol Oncol Clin North Am 29, 271–288, ix, doi: 10.1016/j.hoc.2014.10.003 (2015). 25836934

[b14] Maulard-Durdux C. . Postoperative radiation therapy in 26 patients with invasive transitional cell carcinoma of the upper urinary tract: no impact on survival? J Urol 155, 115–117 (1996). 7490805

[b15] Catton C. N. . Transitional cell carcinoma of the renal pelvis and ureter: Outcome and patterns of relapse in patients treated with postoperative radiation. Urol Oncol 2, 171–176 (1996). 21224165 10.1016/s1078-1439(96)00095-6

[b16] Jwa E. . Adjuvant radiotherapy for stage III/IV urothelial carcinoma of the upper tract. Anticancer Res 34, 333–338 (2014). 24403484

[b17] Chen B. . Radiotherapy may improve overall survival of patients with T3/T4 transitional cell carcinoma of the renal pelvis or ureter and delay bladder tumour relapse. BMC Cancer 11, 297, doi: 10.1186/1471–2407–11–297 (2011). 21756352 PMC3155495

[b18] Jang N. Y., Kim I. A., Byun S. S., Lee S. E. & Kim J. S. Patterns of failure and prognostic factors for locoregional recurrence after radical surgery in upper urinary tract transitional cell carcinoma: implications for adjuvant radiotherapy. Urol Int 90, 202–206, doi: 10.1159/000343729 (2013). 23257513

[b19] Hall M. C., Womack J. S., Roehrborn C. G., Carmody T. & Sagalowsky A. I. Advanced transitional cell carcinoma of the upper urinary tract: patterns of failure, survival and impact of postoperative adjuvant radiotherapy. J Urol 160, 703–706 (1998). 9720526 10.1016/S0022-5347(01)62763-0

[b20] Czito B., Zietman A., Kaufman D., Skowronski U. & Shipley W. Adjuvant radiotherapy with and without concurrent chemotherapy for locally advanced transitional cell carcinoma of the renal pelvis and ureter. J Urol 172, 1271–1275 (2004). 15371822 10.1097/01.ju.0000137910.38441.8a

[b21] Chou Y. H. & Huang C. H. Unusual clinical presentation of upper urothelial carcinoma in Taiwan. Cancer 85, 1342–1344 (1999). 10189140

[b22] Li W. M. . Oncologic outcomes following three different approaches to the distal ureter and bladder cuff in nephroureterectomy for primary upper urinary tract urothelial carcinoma. Eur Urol 57, 963–969, doi: 10.1016/j.eururo.2009.12.032 (2010). 20079965

[b23] Wang S. M. . Increased upper and lower tract urothelial carcinoma in patients with end-stage renal disease: a nationwide cohort study in Taiwan during 1997–2008. Biomed Res Int 2014, 149750, doi: 10.1155/2014/149750 (2014). 25025033 PMC4084494

[b24] Lin C. S. . Hemodialysis Is Associated With Increased Peripheral Artery Occlusive Disease Risk Among Patients With End-Stage Renal Disease: A Nationwide Population-Based Cohort Study. Medicine (Baltimore) 94, e1164, doi: 10.1097/MD.0000000000001164 (2015). 26181560 PMC4617093

[b25] Hsieh S. C., Lin I. H., Tseng W. L., Lee C. H. & Wang J. D. Prescription profile of potentially aristolochic acid containing Chinese herbal products: an analysis of National Health Insurance data in Taiwan between 1997 and 2003. Chin Med 3, 13, doi: 10.1186/1749-8546-3-13 (2008). 18945373 PMC2584031

[b26] Cox J. D., Stetz J. & Pajak T. F. Toxicity criteria of the Radiation Therapy Oncology Group (RTOG) and the European Organization for Research and Treatment of Cancer (EORTC). Int J Radiat Oncol Biol Phys 31, 1341–1346, doi: 10.1016/0360-3016(95)00060-C (1995). 7713792

[b27] Levey A. S. . A more accurate method to estimate glomerular filtration rate from serum creatinine: a new prediction equation. Modification of Diet in Renal Disease Study Group. Annals of internal medicine 130, 461–470 (1999). 10.7326/0003-4819-130-6-199903160-00002 10075613

[b28] Levey A. S. & Coresh J. Chronic kidney disease. Lancet 379, 165–180, doi: 10.1016/S0140–6736(11)60178-5 (2012). 21840587

[b29] Chen W. C. . Concurrent cisplatin, 5-fluorouracil, leucovorin, and radiotherapy for invasive bladder cancer. International journal of radiation oncology, biology, physics 56, 726–733 (2003). 10.1016/s0360-3016(03)00124-x 12788178

